# The prevalence of simian malaria in wild long-tailed macaques throughout Peninsular Malaysia

**DOI:** 10.1038/s41598-024-54981-2

**Published:** 2024-03-12

**Authors:** Shahhaziq Shahari, Mohd Lutfi Bin Abdullah, Anis Adlina Binti Isman Rohimly, Norsharina Binti Ashrat, Amirah Amir, Wahib Mohammed Mohsen Atroosh, Mun Yik Fong, Yee Ling Lau

**Affiliations:** 1 Department of Parasitology, Faculty of Medicine, Universiti Malaya, 50603 Kuala Lumpur, Malaysia; 2 National Wildlife Forensic Laboratory, Ex-Situ Conservation Division, Department of Wildlife and National Parks Peninsular Malaysia, 56100 Kuala Lumpur, Malaysia

**Keywords:** Zoology, Biodiversity, Ecological epidemiology, Population dynamics, Parasitology, Pathogens, Policy and public health in microbiology, Malaria, Parasitic infection, Risk factors

## Abstract

The parasite *Plasmodium knowlesi* has been the sole cause of malaria in Malaysia from 2018 to 2022. The persistence of this zoonotic species has hampered Malaysia’s progress towards achieving the malaria-free status awarded by the World Health Organisation (WHO). Due to the zoonotic nature of *P. knowlesi* infections, it is important to study the prevalence of the parasite in the macaque host, the long-tailed macaque (*Macaca fascicularis*). Apart from *P. knowlesi*, the long-tailed macaque is also able to harbour *Plasmodium cynomolgi, Plasmodium inui*, *Plasmodium caotneyi* and *Plasmodium fieldi.* Here we report the prevalence of the 5 simian malaria parasites in the wild long-tailed macaque population in 12 out of the 13 states in Peninsular Malaysia using a nested PCR approach targeting the *18s ribosomal RNA* (*18s rRNA*) gene. It was found that all five *Plasmodium* species were widely distributed throughout Peninsular Malaysia except for states with major cities such as Kuala Lumpur and Putrajaya. Of note, Pahang reported a malaria prevalence of 100% in the long-tailed macaque population, identifying it as a potential hotspot for zoonotic transmission. Overall, this study shows the distribution of the 5 simian malaria parasite species throughout Peninsular Malaysia, the data of which could be used to guide future malaria control interventions to target zoonotic malaria.

## Introduction

Malaria is still a significant health burden in many countries around the world with 249 million cases reported globally and 608,000 deaths in 2022^1^. This disease is transmitted by *Anopheles* mosquitoes and is primarily caused by six *Plasmodium* species, which are *Plasmodium falciparum*, *Plasmodium vivax*, *Plasmodium malariae*, *Plasmodium ovale curtisi*, *Plasmodium ovale wallikeri* and *Plasmodium knowlesi.* In Malaysia, from the years 2018 to 2022, *P. knowlesi* has been the sole cause of all indigenous malaria cases. The persistence of *P. knowlesi* cases in Malaysia, accounting for 2505 cases and 9 deaths in 2022 alone, has prevented Malaysia from achieving the malaria-free status^1^ by the World Health Organisation.

*Plasmodium knowlesi* is considered a zoonotic malaria species as the parasite originally resides in a macaque host. The currently known hosts of *P. knowlesi* are the long-tailed macaque (*Macaca fasicicularis*), pig-tailed macaque (*Macaca nemestrina*), banded-leaf monkey (*Presbytis melolophus*)^2^ and stumped-tailed macaque (*Macaca arctoides*)^3^. Thus, the transmission of *P. knowlesi* is a complex interaction between the macaque hosts, the Anopheline vectors and humans. It has been hypothesised that urbanisation and deforestation has resulted in increased interactions between humans, macaques and the Anopheline vectors, accounting for the rise of *P. knowlesi* cases over the recent years^4^. Thus, it is important to be able to identify the prevalence of *P. knowlesi* in the macaque hosts as these are the major reservoirs of the parasite.

Apart from *P. knowlesi*, there are other *Plasmodium* species that are able to cause zoonotic infections in humans, four of which are found in wild macaques in Malaysia. The macaques, *M. fascicularis* and *M. nemestrina*, are known to harbour 5 *Plasmodium* species, which are *P. knowlesi*, *Plasmodium cynomolgi*, *Plasmodium inui*, *Plasmodium coatneyi* and *Plasmodium fieldi*. Since the first reported natural human infection of *P. cynomolgi* in 2014^5^, there has been increasing reports of human *P. cynomolgi* infections throughout South East Asia, mostly as asymptomatic cases^6–11^. This suggests that *P. cynomolgi* could also be an emerging zoonotic threat, which needs to be further investigated. Recent reports have implied that *P. inui* too is capable of infecting humans under natural conditions^12–14^. Furthermore, surveillance studies using PCR-based methods in Thailand and indigenous communities in Malaysia, show that potentially *P. coatneyi* and *P. fieldi* could also be infecting humans^12,14^. Overall, the evidence suggests that all 5 of the common simian malaria parasites found in wild macaques in Malaysia should be considered zoonotic infectious agents in humans. Thus, it would be valuable to understand the prevalence of these species in the wild macaque population to be able to identify the zoonotic potential of these parasites.

The long-tailed macaque (*Macaca fascicularis*) is the most common macaque found in Peninsular Malaysia with an estimated population size of 133,403 in the year 2011^15^. This species is also highly anthropophilic, resulting in increased interactions between *M. fascicularis* and humans. Microsatellite analysis in Borneo Malaysia have suggested that the majority of *P. knowlesi* human cases are caused by spill-over infections from the *M. fascicularis* population rather than the *M. nemestrina* population^16^. Thus, highlighting the importance of studying *Plasmodium* species in wild *M. fascicularis* populations.

Malaysia is divided into two regions, Peninsular Malaysia and Malaysia Borneo. A recent study has reported the prevalence of the 5 simian malaria parasites throughout Sarawak, Malaysia Borneo^17^. Although similar simian malaria surveillance studies have been conducted in Peninsular Malaysia, most have only focused on a small number of locations throughout Peninsular Malaysia^18–21^. Here, we determined the prevalence and distribution of *P. knowlesi* and the other simian malaria parasites in the *M. fascicularis* population throughout Peninsular Malaysia, by collecting *M. fascicularis* blood samples from 12 out of the 13 states in Peninsular Malaysia, which were then screened using a nested PCR approach.

## Methods

### Ethics and sample collection

Macaque blood samples were collected as part of a Wildlife Disease Surveillance Programme (WSDP), by the Department of Wildlife and National Parks (DWNP). The permission to conduct this research was obtained from Department of Wildlife and National Parks with the reference no. W-00256-15-19. The protocol for macaque sampling was approved by the University of California, Davis IACUC (Protocol number: 16048) and ethical approval for the use of macaque blood for malaria screening was approved by the Institutional Animal Care and Use Committee Universiti Malaya (UM IACUC) (Reference number: M/06122019/25022019-01/R). All methods were performed in accordance with relevant guidelines and regulations.

Long-tailed macaques were caught using baited traps and anesthetised using a ketamine/xylazine mixture (5 mg/kg of 100 mg/mL ketamine and 100 mg/mL xylazine) via the intramuscular route using a 21G needle by an attending veterinarian from the DWNP. The macaques were sexed and aged by the attending veterinarian, where the age of the macaques was determined by canine and nipple development in males and females respectively. Blood was withdrawn from the macaques with volumes ranging from 2 to 10 mL. This was based on the body weight of the macaques and blood was collected into lithium-heparin blood tubes using a 21G needle attached to a 5 mL syringe (Terumo, Philippines). Macaque blood samples were withdrawn at the trap sites. Whole blood samples were then frozen at − 20 °C until further use. This study was performed in concordance with the Animal Research: Reporting of In Vivo Experiments (ARRIVE) guidelines.

**Figure 1:**
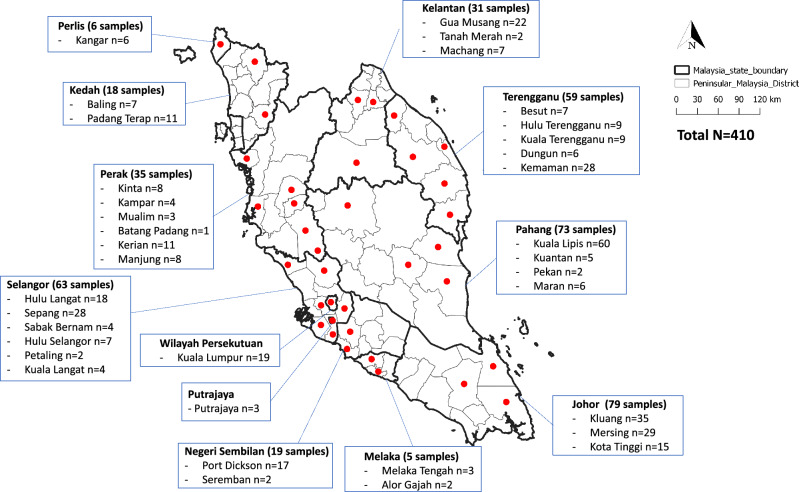
Map of sampling locations (Left) with respective sample sizes (Right). Red dots show districts in which *M. fascicularis* were caught. The map of Peninsular Malaysia was created by the author using QGIS software version 3.6.3 with basemap shapefile modified from the original source on which the data had been plotted (https://data.humdata.org/dataset/cod-ab-mys).

A minimum sample size of 384 macaques was estimated using the equation from Daniel 1999^22^. In total, 410 *M. fascicularis* were caught in 12 states throughout Peninsular Malaysia (Fig. 1), with 62.1% (255/410) of the macaques being male and the majority of the macaques, 55.8% (229/410), being adults (Supplementary Table 1). All *M. fascicularis* sampled were wild macaques. Dates and respective sample sizes for each state are as follows:

Johor (*n* = 79, June 2019 to September 2020), Selangor (*n* = 63, October 2019 to June 2022), Pahang (*n* = 73, October 2019 to September 2022), Melaka (*n* = 5, November 2019), Kelantan (*n* = 31, February 2020), Kedah (*n* = 18, March 2020), Perak (*n* = 35, October 2020 to June 2022), Perlis (*n* = 6, February 2021), Negeri Sembilan (*n* = 19, August 2021 to September 2021), Terengganu (*n* = 59, June 2022 to September 2022), Kuala Lumpur (*n* = 19, June 2022 to September 2022), Putrajaya (*n* = 3, June 2022). For exact coordinates and sample sizes see Supplementary Data (ESRI: 102062 Kertau RSO Malaya Meters format).

### DNA extraction

DNA was extracted from 100 μL of whole blood using the DNeasy® Blood and Tissue kit (QIAGEN) according to the manufacturer’s protocol. A total of 100 μL was eluted such that 1 μL of eluted DNA represented 1 μL of whole blood. DNA was stored at − 20 °C until further use. With each extraction batch, a DNA extraction negative control was added where 100 μL of phosphate buffered saline (PBS) was used as a sample.

### Nested PCR assay

Four microliter of eluted DNA sample was used for screening. Samples were initially screened using a *Plasmodium*-specific nested PCR targeting the *18s rRNA* gene as described previously^23^. Samples that were positive for *Plasmodium* parasites were then screened using a species-specific nested PCR targeting the *18s rRNA* gene of *P. knowlesi*, *P. cynomolgi*, *P. inui*, *P. coatneyi* and *P. fieldi* as described previously^24^. Each batch of PCR included a DNA extraction negative control, a no template control and positive controls for each species. Positive controls came in the form of samples previously confirmed for each species with nested PCR^20^. For quality assurance, a set of random samples were taken and PCR repeated to confirm results. For representative gel images see Supplementary Figs. 1–3.

## Results

Of the 410 *M. fascicularis* that were caught, 49.8% (204/410) were positive for malaria parasites. Pahang had the highest prevalence of malaria positive macaques, where 100% (73/73) were positive. This was followed by Johor (54.4%, 43/79), Terengganu (52.5%, 31/59), Selangor (49.2%, 31/63), Kelantan (48.4%, 15/31), Perak (25.7%, 9/35), Perlis (16.7%, 1/6) and Negeri Sembilan (5.3%, 1/19). All macaques caught from Melaka, Kedah, Kuala Lumpur and Putrajaya were negative (Table 1).

**Table 1:** Prevalence of malaria parasites in wild *M. fascicularis* from Peninsular Malaysia.

State	Host species	PCR positive	Prevalence (%)
Johor	*Macaca fascicularis* (n = 79)	43	54.4
Selangor	*Macaca fascicularis* (n = 63)	31	49.2
Pahang	*Macaca fascicularis* (n = 73)	73	100.0
Melaka	*Macaca fascicularis* (n = 5)	0	0.0
Kelantan	*Macaca fascicularis* (n = 31)	15	48.4
Kedah	*Macaca fascicularis* (n = 18)	0	0.0
Perak	*Macaca fascicularis* (n = 35)	9	25.7
Perlis	*Macaca fascicularis* (n = 6)	1	16.7
Negeri Sembilan	*Macaca fascicularis* (n = 19)	1	5.3
Terengganu	*Macaca fascicularis* (n = 59)	31	52.5
Putrajaya	*Macaca fascicularis* (n = 3)	0	0.0
Kuala Lumpur	*Macaca fascicularis* (n = 19)	0	0.0
Total	*Macaca fascicularis* (N = 410)	204	49.8

Throughout Peninsular Malaysia, *P. inui* was the most prevalent at 37.1%, followed by *P. fieldi* (32.9%), *P. cynomolgi* (31.2%), *P. coatneyi* (31.0%) and *P. knowlesi* (29.0%) (Table 2). It was noted that the predominant species varied between states where *P. inui* was the most prevalent for Johor, Pahang, Perak and Perlis, whilst *P. cynomolgi* was the most prevalent for Selangor, Kelantan and Negeri Sembilan (Table 2). The majority of infected macaques were infected by 2 or more *Plasmodium* species, where the most common infection type was an infection with all 5 simian *Plasmodium* species (Table 3). However, dual infections were the most common in Selangor, Kelantan, Perlis and Negeri Sembilan, whilst quadruple infections were the most common in Johor (Supplementary Table 2). This indicates that the species distribution and infection type of simian malaria parasites in the wild *M. fascicularis* population in Peninsular Malaysia is highly location dependant.

**Table 2:** Distribution of *Plasmodium* species in infected *M. fascicularis* by state.

Species	State infected (prevalence)								Total (N = 410)
	JOH (n = 79)	SEL (n = 63)	PAH (n = 73)	KEL (n = 31)	PRK (n = 35)	PLS (n = 6)	NGS (n = 19)	TER (n = 58)	
*Pk*	25 (31.6)	18 (28.6)	45 (61.6)	6 (19.4)	4 (11.4)	0 (0)	0 (0)	21 (35.6)	119 (29.0)
*Pcy*	23 (29.1)	22 (34.9)	51 (69.9)	10 (32.3)	1 (2.9)	0 (0)	1 (5.3)	20 (33.9)	128 (31.2)
*Pin*	34 (43.0)	13 (20.6)	65 (89.0)	6 (19.4)	7 (20.0)	1 (16.7)	0 (0)	26 (44.1)	152 (37.1)
*Pct*	24 (30.4)	11 (17.5)	62 (84.9)	4 (12.9)	6 (17.1)	0 (0)	0 (0)	20 (33.9)	127 (31.0)
*Pfi*	20 (35.3)	17 (27.0)	53 (72.6)	9 (29.0)	7 (20.0)	1 (16.7)	1 (5.3)	27 (45.8)	135 (32.9)

*Pk P. knowlesi*, *Pcy P. cynomolgi*, *Pin P. inui*, *Pct P. coatneyi*, *Pfi P. fieldi*, *JOH* Johor, *SEL* Selangor, *PAH* Pahang, *KEL* Kelantan, *PRK* Perak, *PLS* Perlis, *NGS* Negeri Sembilan, *TER* Terengganu.

**Table 3:** Summary of infection types in wild *M. fascicularis* throughout Peninsular Malaysia.

Location	Infection type	Plasmodium species	Infected	Total	Percentage (%) (N = 410)
Peninsular Malaysia (N = 410)	Mono	Pk	10	25	6.1
		Pcy	8		
		Pin	2		
		Pct	5		
	Dual	Pk + Pcy	10	47	11.5
		Pk + Pin	1		
		Pk + Pfi	1		
		Pcy + Pin	5		
		Pcy + Pct	4		
		Pcy + Pfi	7		
		Pin + Pct	6		
		Pin + Pfi	13		
	Triple	Pk + Pcy + Pin	1	36	8.8
		Pk + Pcy + Pct	1		
		Pk + Pcy + Pfi	2		
		Pk + Pin + Pct	3		
		Pk + Pin + Pfi	7		
		Pk + Pct + Pfi	1		
		Pcy + Pin + Pct	1		
		Pcy + Pin + Pfi	5		
		Pcy + Pct + Pfi	1		
		Pin + Pct + Pfi	14		
	Quadruple	Pk + Pcy + Pin + Pct	12	46	11.2
		Pk + Pcy + Pin + Pfi	5		
		Pk + Pcy + Pct + Pfi	2		
		Pk + Pin + Pct + Pfi	13		
		Pcy + Pin + Pct + Pfi	14		
	Quintuple	Pk + Pcy + Pin + Pct + Pfi	50	50	12.2

*Pk P. knowlesi*, *Pcy P. cynomolgi*, *Pin P. inui*, *Pct P. coatneyi*, *Pfi P. fieldi.*

## Discussion

Overall, this study determined the prevalence of the five simian malaria parasites in the wild *M. fascicularis* population of Peninsular Malaysia to be 49.8%. This is comparable to a previous study, with an earlier sampling period of 2016 to 2019, that looked at only seven states in Peninsular Malaysia and found a prevalence of 48.2%^21^. However, one limitation in comparing between these studies is that the methodology between the studies were not identical. The study conducted by Yusuf et al. performed PCR only on samples that were first confirmed positive via microscopy. Considering that microscopy is less sensitive than PCR, the prevalence of malaria in the *M. fascicularis* population could have been underestimated^21^. Notably, Pahang reported a prevalence of 100% with most of the macaques being infected with all 5 simian *Plasmodium* species. This indicates that Pahang is a potential hotspot for zoonotic malaria transmission and indeed, many of the *P. knowlesi* human cases in Peninsular Malaysia have been reported from Pahang^25^. Previous studies across a sampling period of 2016^20^ and 2016 to 2019^21^ reported a prevalence of 88.2% (30/34) and 93.6% (176/288) in Pahang, respectively. This could suggest that there could be an increasing trend of simian malaria prevalence in Pahang itself. Thus, although the overall trend of simian malaria prevalence throughout Peninsular Malaysia has not changed much over the years, there may be variations within specific states. This is evident in the differences found in species prevalence and infection types seen between the different states (Table 2, Supplementary Table 2). However, this study and previous studies investigating the prevalence of malaria in the macaque population have primarily been cross-sectional studies. Thus, it is difficult to infer this trend, which identifies the need for longitudinal-based studies.

*Plasmodium knowlesi* was the least prevalent (29.0%) of the 5 simian malaria species in the macaque population, but is the species responsible for the majority of human cases in Malaysia^1^. This highlights the complexity of zoonotic transmission to humans as different species may vary in transmissibility, exposure and susceptibility to humans. Spatial analysis of *P. knowlesi* human cases in Peninsular Malaysia from 2011 to 2018 revealed that *P. knowlesi* cases tended to cluster around the Kelantan-Pahang border, particularly in the Gua Musang (Kelantan) and Kuala Lipis (Pahang) districts^25^. Here we report a *P. knowlesi* macaque prevalence of 19.4% and 61.6% for Kelantan and Pahang, respectively. Notably, Johor (31.6%), Selangor (28.6%) and Terengganu (35.6%) reported a higher *P. knowlesi* prevalence than Kelantan. Thus, although prevalence of the parasite in the macaque host is an important risk factor^26^, this cannot be taken in isolation. Males with outdoor-based occupations such as plantation workers seem to be most at risk to *P. knowlesi* infection^26^. Furthermore, the tendency of *P. knowlesi* vectors to bite outdoors and have peak biting times earlier in the evening^27^ make interventions such as insecticide treated bed nets and indoor residual spraying ineffective to reduce *P. knowlesi* cases^28^. Thus, it would be important to combine data from the macaque population, mosquito behavioural studies and human cases in order to design a one health approach for interventions against *P. knowlesi.*

The prevalence of *P. cynomolgi* was high (> 30%) in Selangor, Pahang, Kelantan and in particular Terengganu, which is where the first reported *P. cynomolgi* human case was discovered in 2014^5^. Since then, surveillance studies in Cambodia, Thailand and Malaysia have detected numerous accounts of *P. cynomolgi* cases in humans, most of which were asymptomatic^8,11,12,14^. Furthermore, case studies have reported *P. cynomolgi* infections in travellers returning from South East Asia^9^. This implies that further research into the epidemiology of *P. cynomolgi* is needed to ensure that *P. cynomolgi* does not become a major public health threat in the future. However, the identification of *P. cynomolgi* in patients can be difficult. This is due to the similarities between *P. cynomolgi* and *P. vivax* resulting in misidentification microscopically^2^ and in some cases even via molecular diagnosis^5^.

*Plasmodium inui* was the most prevalent species found in the wild *M. fascicularis* population (37.1%), with Pahang reporting a prevalence of 89.0%. This suggests that the risk of exposure of *P. inui* to humans could be high as *P. inui* was also the most prevalent species found in *Anopheles* mosquitoes sampled throughout Peninsular Malaysia^29^. Experimental infections with *P. inui* into humans have been shown to result in symptomatic febrile infections^30^ and a total of 24 human cases have been reported from Malaysia and Thailand^12–14^. The 5 human cases reported from Pahang and Melaka in Peninsular Malaysia and Sarawak in Malaysia Borneo were from surveillance studies rather than patients suggesting that these cases were all asymptomatic individuals^12,13^. Conversely, the 19 *P. inui* human cases reported from Thailand^14^, were all symptomatic patients reporting into a malaria clinic. However, 18/19 patients had coinfections with *P. vivax* and/or *P. falciparum*. Thus, it is difficult to identify whether *P. inui* contributed to the presentation of the symptoms. One patient was mono-infected with *P. inui* suggesting that *P. inui* infections in humans can result in symptomatic presentation, though rarely. Thus, it is possible that *P. inui* human infections may be currently underestimated due to the high likelihood of asymptomatic presentation. This highlights the need for large scale surveillance studies to identify the burden of *P. inui* infections in humans, especially in areas where *P. inui* is prevalent in the wild macaque hosts.

Evidence to suggest that *P. coatneyi* and *P. fieldi* infecting humans is still very preliminary. Although *P. coatneyi* and *P. fieldi* was found to be widely distributed across Peninsular Malaysia, previous experimental infections of *P. coatneyi* and *P. fieldi* to humans were unsuccessful^2,31^. However, two surveillance studies, have found *P. coatneyi*- and *P. fieldi-*positive patients via molecular techniques in Malaysia^12^ and Thailand^14^, respectively. For *P. coatneyi*, 3 patients from an indigenous community in Perak, Malaysia, were found to be positive. However, screening of the same samples in two different laboratories found inconsistent findings^12^. For, *P. fieldi*, 3 positive patients were found in Yala province in Southern Thailand, which borders Malaysia. Although these samples were microscopically positive, all three patients were coinfected with *P. inui* and *P. vivax*^14^. Overall, these studies provide preliminary evidence to suggest that *P. coatneyi* and *P. fieldi* can cause zoonotic infections in humans. Furthermore, these infections may be present at submicroscopic levels, suggesting screening using larger blood volumes, as has been found with both *P. cynomolgi*^8^ and *P. inui*^13^. Considering that the prevalence of *P. coatneyi* and *P. fieldi* in this study was 31.0% and 32.9% respectively, the zoonotic threat of these species in Peninsular Malaysia has yet to be determined.

One of the major limitations of this study is that samples were collected primarily through convenience sampling. Monkey baited traps were set up by the DWNP based on conflict case reports. This resulted in variable sampling periods across the states. Thus, one possibility is that the differences in prevalence between states could be due to temporal variations in transmission (e.g. during the monsoon vs dry seasons). This study has identified states of high prevalence such as Pahang (100%), Johor (54.4%), Terengganu (52.5%), Selangor (49.2%) and Kelantan (48.4%). Thus, to overcome the limitations due to temporal variation, these states could be used as sites for future longitudinal-based studies. Another limitation is that the reliance on convenience sampling has resulted in small sample sizes for Melaka (*n* = 5), Perlis (*n* = 6) and Putrajaya (*n* = 3). Thus, for these three states, it is difficult to conclude the prevalence of malaria in the *M. fascicularis* population. Thus, it would be beneficial for future studies to include these states with a higher sample size.

In conclusion, this study showed that the *P. knowlesi*, *P. cynomolgi*, *P. inui*, *P. coatneyi* and *P. fieldi* are widely distributed across Peninsular Malaysia in the *M. fascicularis* population. Although *P. knowlesi* was the least prevalent among the 5 species, it is the species that is responsible for the majority of symptomatic cases in humans^25^. In contrast, *P. cynomolgi* and *P. inui* although more prevalent in the macaque population, have comparatively fewer human cases and mostly result in asymptomatic infections^8,11–14^. This highlights that *P. knowlesi* is better adapted to grow in humans in comparison to the other simian malaria species^2^. Thus, an assessment of zoonotic risk should include both the prevalence in the macaque host as well as the adaptability of the parasite species to humans, among other factors. From a medical standpoint, all 5 simian malaria species can currently be treated with standard antimalarial drugs with no signs of drug resistance^5,14^. This is likely due to the absence of drug pressure on these populations in the main reservoir hosts, the macaques. As *P. knowlesi* is the main cause of malaria in Malaysia, residents and travellers to the different states should be made aware of the risk of potentially acquiring an infection in these states. Furthermore, as more human cases of *P. cynomolgi*, *P. inui* and potentially *P. coatneyi* and *P. fieldi* emerge, it would be beneficial for health care personnel to be aware of regions where these parasites are prevalent in the macaque population. This could help elucidate the zoonotic potential of these simian malaria species as zoonotic malaria may pose a threat to the goal of global malaria elimination.

### Supplementary Information

Supplementary Information 1.

Supplementary Information 2.

Supplementary Table 2.

## Data Availability

The datasets generated during and/or analysed during the current study are available from the corresponding author on reasonable request.
