# Supplementary material for: The prevalence of simian malaria in wild long-tailed macaques throughout Peninsular Malaysia
Source: Sci Rep. 2024 Mar 12;14:6023. doi: 10.1038/s41598-024-54981-2 (PMC10933401; doi:10.1038/s41598-024-54981-2)
Supplement: Supplementary file 2 — Supplementary Information 2. [file 41598_2024_54981_MOESM2_ESM.pdf]

Supplementary Figures

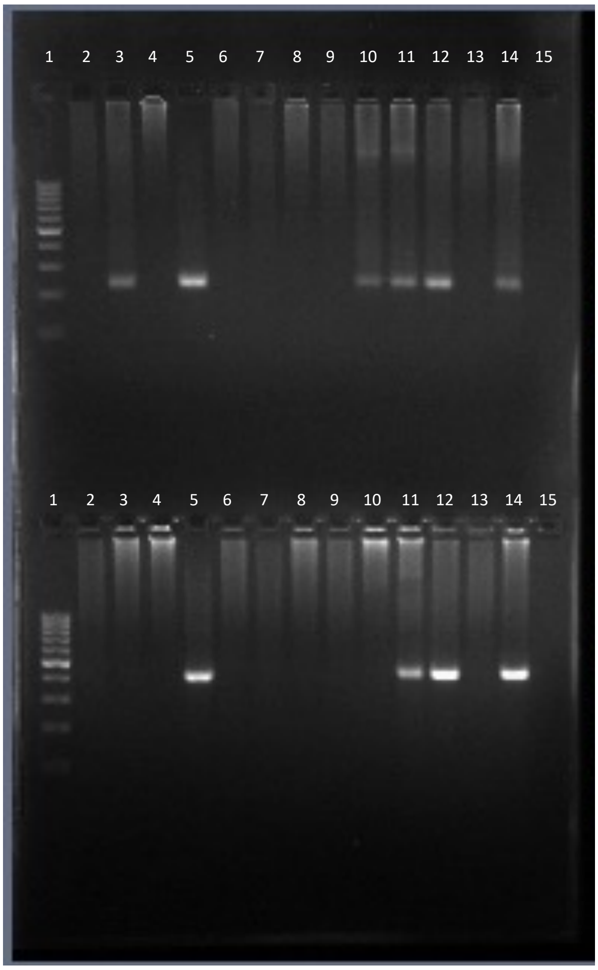

<u>Genus screening (rPLU3/rPLU4)</u>

1: GeneRuler 100bp
2: KTLT001
3: KTLT002
4: KTLT003
5: KTLT004
6: KTLT005
7: DNA extraction negative control
8: KTLT006
9: KTLT007
10: KTLT008
11: KTLT009
12: KTLT010
13: No template control
14: Positive control
15: Blank lane

<u>*P. knowlesi* screening (PkF1140/PkR1550)</u>

1: GeneRuler 100bp
2: KTLT001
3: KTLT002
4: KTLT003
5: KTLT004
6: KTLT005
7: DNA extraction negative control
8: KTLT006
9: KTLT007
10: KTLT008
11: KTLT009
12: KTLT010
13: No template control
14: Positive control
15: Blank lane

Supplementary Figure 1: Representative gel image for genus screening (primers: rPLU3/rPLU4) and *P. knowlesi* screening (primers: PkF1140/PkR1550) using nested PCR for sample KTLT001 to KTLT010.

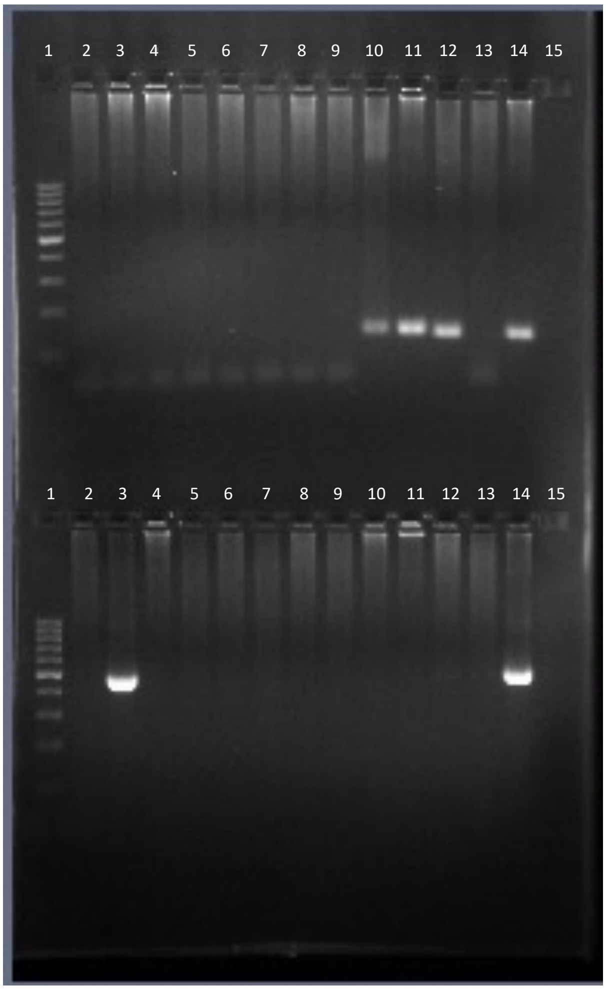

***P. cynomolgi* screening (CY2F/CY4R)**

1: GeneRuler 100bp
2: KTLT001
3: KTLT002
4: KTLT003
5: KTLT004
6: KTLT005
7: DNA extraction negative control
8: KTLT006
9: KTLT007
10: KTLT008
11: KTLT009
12: KTLT010
13: No template control
14: Positive control
15: Blank lane

***P. coatneyi* screening (PctF1/PctR1)**

1: GeneRuler 100bp
2: KTLT001
3: KTLT002
4: KTLT003
5: KTLT004
6: KTLT005
7: DNA extraction negative control
8: KTLT006
9: KTLT007
10: KTLT008
11: KTLT009
12: KTLT010
13: No template control
14: Positive control
15: Blank lane

Supplementary Figure 2: Representative gel image for *P. cynomolgi* screening (primers: CY2F/CY4R) and *P. coatneyi* screening (primers: PctF1/PctR1) using nested PCR for sample KTLT001 to KTLT010.

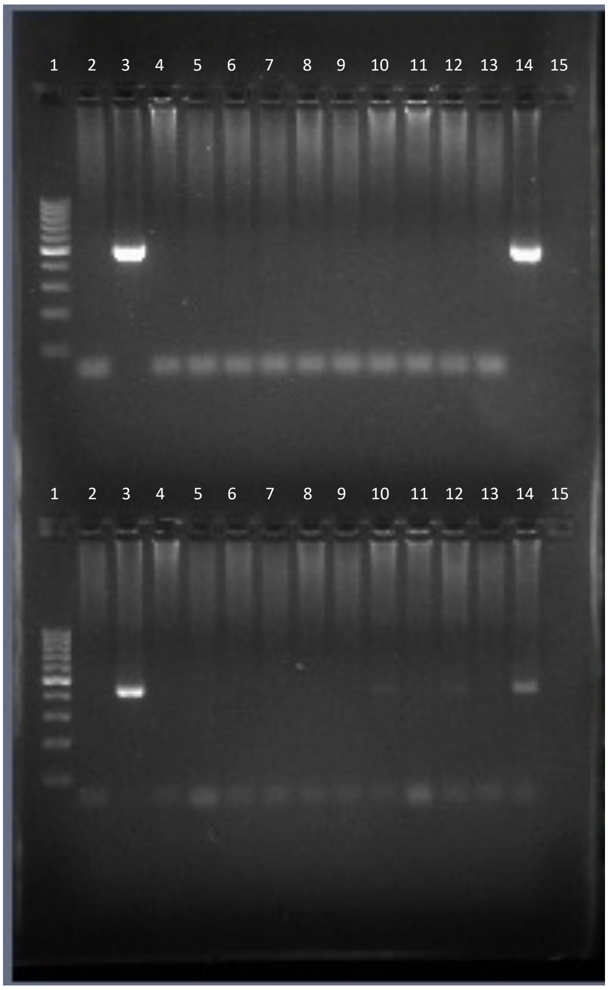

P. inui screening (PinF2/INAR3)
1: GeneRuler 100bp
2: KTLT001
3: KTLT002
4: KTLT003
5: KTLT004
6: KTLT005
7: DNA extraction negative control
8: KTLT006
9: KTLT007
10: KTLT008
11: KTLT009
12: KTLT010
13: No template control
14: Positive control
15: Blank lane

P. fieldi screening (PfldF1/PfldR2)
1: GeneRuler 100bp
2: KTLT001
3: KTLT002
4: KTLT003
5: KTLT004
6: KTLT005
7: DNA extraction negative control
8: KTLT006
9: KTLT007
10: KTLT008
11: KTLT009
12: KTLT010
13: No template control
14: Positive control
15: Blank lane

Supplementary Figure 3: Representative gel image for *P. inui* screening (primers: PinF2/INAR3) and *P. fieldi* screening (primer PfldF1/PfldR2) using nested PCR for sample KTLT001 to KTLT010.

Supplementary Table 1: Demographic data of wild macaques sampled.

State	Host species	Sex			Age				
		Male	Female	Not recorded	Adult	Subadult	Juvenile	Infant	Not recorded
Johor (n=79)	*Macaca fascicularis*	56	23	0	51	13	14	1	0
Selangor (n=63)	*Macaca fascicularis*	46	17	0	28	0	27	8	0
Pahang (n=73)	*Macaca fascicularis*	41	24	8	35	12	18	0	8
Melaka (n=5)	*Macaca fascicularis*	5	0	0	1	3	1	0	0
Kelantan (n=31)	*Macaca fascicularis*	19	12	0	17	3	11	0	0
Kedah (n=18)	*Macaca fascicularis*	16	2	0	6	7	5	0	0
Perak (n=35)	*Macaca fascicularis*	15	20	0	29	0	6	0	0
Perlis (n=6)	*Macaca fascicularis*	4	2	0	4	0	2	0	0
Negeri Sembilan (n=19)	*Macaca fascicularis*	12	7	0	19	0	0	0	0
Terengganu (n=59)	*Macaca fascicularis*	30	15	14	24	0	21	0	14
Putrajaya (n=3)	*Macaca fascicularis*	1	2	0	0	0	3	0	0
Kuala Lumpur (n=19)	*Macaca fascicularis*	10	9	0	15	0	4	0	0
**Total (N=410)**	***Macaca fascicularis***	**255**	**133**	**22**	**229**	**38**	**112**	**9**	**22**
